# A case-control study on occupational risk factors for sino-nasal cancer

**DOI:** 10.1136/oem.2008.041277

**Published:** 2009-01-19

**Authors:** A d’Errico, S Pasian, A Baratti, R Zanelli, S Alfonzo, L Gilardi, F Beatrice, A Bena, G Costa

**Affiliations:** 1 Epidemiology Unit, Piedmont Region, Grugliasco, Italy; 2 Occupational Medicine Department, Savigliano, Italy; 3 Occupational Health and Safety Department, Asti, Italy; 4 Occupational Health and Safety Department, Saluzzo, Italy; 5 Documentation Center for Health Promotion, Grugliasco, Italy; 6 Otolaryngology (ENT) Unit, Giovanni Bosco Hospital, Turin, Italy; 7 University of Turin, Department of Public Health, Turin, Italy

## Abstract

**Objectives:**

Sino-nasal cancer has been consistently associated with exposure to wood dust, leather dust, nickel and chromium compounds; for other occupational hazards, the findings are somewhat mixed. The aim of this study was to investigate the risk of sino-nasal epithelial cancer (SNEC) by histological type with prior exposure to suspected occupational risk factors and, in particular, those in metalworking.

**Methods:**

Between 1996 and 2000, incident cases were collected on a monthly basis from hospitals throughout the Piedmont region of Italy by the regional Sino-nasal Cancer Registry. A questionnaire on occupational history, completed by 113 cases and 336 hospital controls, was used to assign exposure to occupational hazards. The relationship between SNEC and cumulative exposure to these hazards was explored using unconditional logistic regression to statistically adjust for age, sex, smoking and co-exposures, allowing for a 10-year latency period.

**Results:**

The risk of adenocarcinoma was significantly increased with ever-exposure to wood dust (odds ratio; OR = 58.6), and to leather dust (OR = 32.8) and organic solvents (OR = 4.3) after controlling for wood dust, whereas ever-exposure to welding fumes (OR = 3.7) and arsenic (OR = 4.4) significantly increased the risk for squamous cell carcinoma. For each of these hazards, a significant increasing trend in risk across ordered cumulative exposure categories was found and, except for arsenic, a significantly increased risk with ever-exposure at low intensity. Treating cumulative exposure on a continuous scale, a significant effect of textile dusts was also observed for adenocarcinoma. For a mixed group of other histological types, a significant association was found with wood dust and organic solvents.

**Conclusions:**

Some occupational risk factors for SNEC were confirmed, and dose–response relationships were observed for other hazards that merit further investigation. The high risk for adenocarcinoma with low-intensity exposure to wood dust lends support for a reduction in the occupational threshold value.

Malignant tumours of the nose and paranasal sinuses (SNC) are uncommon neoplasms with an incidence of less than one per 100 000 inhabitants in most developed countries.^1^ Over three-quarters of all SNC consist of malignant epithelial tumours, among which the most common histologies are adenocarcinoma and squamous cell carcinoma.^2^

A distinct characteristic of SNC and, in particular, of epithelial tumours is that the major risk factors are workplace hazards. Since the late 1960s, many epidemiological studies have found a high risk of SNC among employees in woodworking and furniture production,^3^^–^5 nickel refining^6^ ^7^ and the manufacture of shoes and other leather products.^8^ ^9^ The association between adenocarcinoma and wood dust exposure, especially hardwood, is well established and contributed to its classification by the International Agency for Research on Cancer (IARC) as definitely carcinogenic to humans.^10^ For other histotypes, the relationship appears to be less consistent and the risk is much lower.^11^ Nickel compounds are acknowledged risk factors for SNC, and are also classified as definitely carcinogenic to humans.^12^ The risks for SNC observed in shoe and leather manufacturing are likely attributable to leather dust exposure, as the risk is especially high among the most heavily exposed workers employed in finishing^8^ and there is evidence of a dose–response effect.^9^ Nonetheless, only the “boot and shoe manufacture and repair” sector has been classified as a group 1 carcinogen and not leather dust itself,^13^ ^14^ as the chemicals used in leather manufacturing may have been responsible for the excess risks. The association with leather dust appears mainly with adenocarcinoma, although an increased risk has been reported for other histologies.^9^ ^13^

**Main messages**

- The risk of any sino-nasal epithelial cancer was significantly related to prior exposure to wood dust, leather dust, organic solvents, welding fumes and arsenic.
- A dose–response relationship was observed for each of the above hazards.
- The hazardous exposures appear to differ by histological type: wood dust, leather dust, organic solvents and textile dusts were risk factors for adenocarcinoma, whereas exposure to welding fumes and arsenic increased the risk for squamous cell carcinoma.
- The risk of adenocarcinoma was also higher among participants who had been exposed to wood or leather dust at low-intensity levels.
- The associations with welding fumes, organic solvents and textile dusts merit further investigation.

**Policy implication**

- The high risk for adenocarcinoma with low-intensity exposure to wood dust supports the need to reduce the current occupational exposure limit for this hazard.

Occupational exposure to hexavalent chromium compounds, also definite human carcinogens, has been consistently linked to SNC.^12^ Increased risks of SNC have also been found, with some inconsistencies, for many other occupations, including farm workers,^15^ textile^4^ ^16^ ^17^ and chemical^18^ workers, bakers and pastry confectioners,^19^ construction workers,^15^ welders^20^ and other metal workers.^21^

The rarity of SNC makes it unlikely that excess risks will be observed in cohort studies; except for a few cohorts consisting mainly of workers employed in the wood and leather industries or in nickel refining, most of the evidence relating SNC to occupational exposures comes from case-control studies in which the risk estimates are often based on small numbers of exposed cases, and the prevalence of exposure to most suspected hazards is low.

The aim of this study was to investigate the risk of sino-nasal epithelial cancer (SNEC) with prior exposure to suspected occupational risk factors, in the Piedmont region of Italy. As metalworking was one of the most common occupations in Piedmont for many decades after the Second World War, the prevalence of exposure to the known or suspected carcinogenic agents to which workers would have been exposed in this industry was sufficiently high to examine the risks by histological types.

## METHODS

Starting in 1996, all Piedmont hospital departments in which SNC could be diagnosed or treated were contacted monthly by the Piedmont SNC Registry, established for the identification of occupational-related SNC cases. By 2000, 154 incident cases of primary SNEC (International Statistical Classification of Diseases and Related Health Problems, 9th revision: 160.0, 160.2–160.5) with histological confirmation had been identified in 22 otolaryngology (ear, nose and throat; ENT) and two maxillo-facial surgery departments. A crude incidence rate of 0.73 SNEC cases per 100 000 inhabitants was estimated using the 2001 Census data for the denominator (1.19 per 100 000 among men and 0.30 per 100 000 among women). Forty-one cases were excluded: three prevalent cases; 36 not interviewed or interviewed using a pilot version of the questionnaire; and two under 30 years old, in consideration of the long latency of SNC tumours.^22^ Among the hospital controls contacted between 1998 and 2002, the non-response rate was 5%, and six were excluded (four not interviewed and two under 30 years old). The 336 controls were recruited from departments of ENT and orthopaedics, frequency matched to cases by age (10-year classes), sex and province of residence.

Study participants completed a 1 h open-ended questionnaire administered by trained interviewers (not blind to case-control status) on job history, demographics and tobacco smoking. For each working period, defined as a continuous period of employment in the same job, participants were asked to report on: company size and type of economic activity; job title; description, frequency and duration of their tasks; size of their work environment, number of people working in it and the tasks performed by them; description of machinery in their work environment; substances used by themselves and others in their proximity; proportion of working hours spent outdoors; presence of dusts, fumes and vapours in their work environment; use of protective devices; and the presence and efficiency of exhaust ventilation systems. All subjects gave their informed consent to participate in this study.

The list of possible risk factors for SNC included arsenic, wood dust, leather dust, nickel compounds, chromium VI and its salts, polycyclic aromatic hydrocarbons (PAHs), welding fumes, oil mists, formaldehyde, flour, cocoa powder, textile dusts, silica, coal dust, paint mists, strong acid mists and organic solvent vapours. Wood dust was not separated into hardwood or softwood as this information was collected only in terms of lifetime occupational exposure.

For each working period of at least 6 months, the probability and intensity of exposure to each hazard were independently rated based on the information provided in the questionnaires, by an occupational epidemiologist and by one of two occupational physicians; in the event of non-concordant ratings (38%), the questionnaire was re-evaluated by a senior industrial hygienist, all of whom were blind to case-control status. In addition, job title (ISCO-68) and economic sector (ISIC-74) codes, assigned by an expert, were used in conjunction with two job-exposure matrices (JEMs) to aid in evaluating some exposures: solvent vapours, arsenic, PAHs, chromium and nickel compounds^23^ ^24^; wood, leather, textile, coal and silica dusts, flour, formaldehyde.^25^ For these hazards, the probability and intensity provided in the JEM by economic sector and job title were adjusted in accordance with information in the questionnaire (historical period and working conditions), expert opinion of the raters and, in some instances, industrial hygiene reports. The probability of exposure was rated on a four-level scale, in which “unexposed” corresponds roughly to less than 10%, “low” to between 10 and 50%, “medium” in the range 50–90% and “high” to a probability above 90%. The intensity rating was either negligible (unexposed), low, medium or high.

A working period was defined by its start and end year in which mid-year was used to calculate the duration of exposure in years. A latency period of 10 years was assumed and, therefore, any exposure occurring within 10 years of diagnosis was excluded. For each hazard, the duration of exposure was weighted by the probability and intensity of exposure, and “cumulative exposure” was obtained by summing the weighted duration over all working periods. As shown in table 1, high-intensity exposures had all been rated as high probability, and medium intensity as either medium or high probability. In selecting the weights, intensity was considered as two levels (low and medium–high) given the sparseness of data. Exposures that had been rated as high probability and either medium or high intensity were weighted at 100%, such that every year of exposure counted toward cumulative exposure. The weight assigned to exposures rated as low probability, all of which were at low intensity, was 30%, the mid-point of the probability range. One year of cumulative exposure, for instance, corresponds to 3.5 years of low-intensity exposure if at low probability, 2 years if at medium and 1.5 years if rated as high probability.

**Table 1:** Years of exposure weighted by probability and intensity equivalent to 1 year of cumulative exposure*

Probability		Intensity			Duration		
		Low	Medium	High			
		Assigned weights			Years of exposure		
10–50%	Low	0.3			3.5		
50–90%	Medium	0.5	0.7		2.0	1.5	
>90%	High	0.7	1.0	1.0	1.5	1.0	1.0

*Assigned weight × years of exposure  =  cumulative exposure.

### Statistical analysis

The relationship between the probability of SNEC and cumulative exposure to occupational hazards was explored using unconditional logistic models to estimate relative risks statistically adjusted for co-exposures; this relationship was examined further by histological type (adenocarcinoma, squamous cell carcinoma and a mixed group of other histotypes). In a preliminary analysis, odds ratios (ORs) for ever-exposure to hazards were adjusted by age and sex, as there was no interaction with sex. The ever-exposed categorisation was defined as cumulative exposure of at least 1 year.

Given the small number of exposed cases and the inherent loss of information in categorising quantitative variables, multivariable models were fitted treating cumulative exposure to all hazards as continuous variables, with the assumption that the multiplicative change in risk for every unit increase in years of exposure is constant over the range of each hazard. The relative risk estimates obtained using unconditional logistic regression to model continuous data from case-control studies have been shown to be relatively robust over the range to which 90% of the population are exposed, even if the data are incorrectly modelled as continuous variables.^26^ The independent variables were sex, age (continuous), smoking status (never, former, current) and those hazards with at least three exposed cases.

In two subsequent analyses, cumulative exposure was defined as categorical variables: (1) dichotomous (ever/never exposed) and (2) polychotomous (three ordered exposure categories — unexposed and two classes chosen according to the range of each hazard in the sample data). Finally, the effect of exposure intensity was evaluated separately, weighting duration of exposure only by probability (0.3, 0.7 and 1.0 for low, medium and high, respectively) and treating intensity as a categorical variable (unexposed, exposed only at low intensity, ever exposed at medium–high intensity).

The same strategy was followed to select a best subset model in each analysis, including an assessment of multicollinearity. Mallows’ Cp statistic, which is a measure of prediction error, was used to choose among all possible regression models with preference given to those with the smallest Cp values. As these models tend to contain the most important predictors and minimise the error in prediction, they were assessed in selecting the final models.^27^ First-order interactions were assessed in the models with categorical exposure variables. All tests were two-sided, for which a significance level of 0.05 was adopted. SAS (version 8) and STATA (version 8) were used to perform the analyses.

The effects of potential confounding variables were considered; a confounder was retained in the model if its removal resulted in a change of 15% or more in any regression coefficient. In the models with categorical variables, confounding by wood dust was assessed by adjusting for it on a continuous scale. The presence of a trend in risk across ordered exposure classes was examined by treating these variables as continuous. Diagnostic checks on model adequacy included verifying that the logit was linear in the continuous independent variables.

The stability of the final regression models was ascertained by restricting the analyses to exposures that had occurred with a probability of at least 50% (medium and high). The final models for adenocarcinoma were also compared with those obtained when the analysis was restricted to those persons who had not been exposed to leather dust, which excluded four cases and two controls. It was not possible to do the same for wood dust, because the remaining cases were too few in number to examine the effects of other hazards.

## RESULTS

Of the 113 cases, 53 were adenocarcinomas (ADs), 37 squamous cell carcinomas (SCCs) and 23 were other histologies (OHs), including 11 undifferentiated, six mucoepidermoid, one neuroendocrin, one basocellular and four unspecified carcinomas (table 2). Cases and controls did not differ significantly in the average number of years worked in ISCO 1-digit occupational groups 5 to 9, which consist mainly of manual workers (32.9 vs 30.1 years, t test: p = 0.14).

**Table 2:** Distribution of sino-nasal epithelial cancer by age, sex and smoking status

	Adenocarcinoma	Squamous cell carcinoma	Other histotypes	Controls
*Age (years)*	No (%)	No (%)	No (%)	No (%)
30–49	3 (5.7)	6 (16.2)	1 (4.4)	51 (15.2)
50–59	4 (7.5)	6 (16.2)	5 (21.7)	62 (18.5)
60–69	26 (49.1)	15 (40.6)	9 (39.1)	109 (32.4)
≥70	20 (37.7)	10 (27.0)	8 (34.8)	114 (33.9)
*Sex*				
Males	45 (84.9)	12 (32.4)	19 (82.6)	234 (69.6)
Females	8 (15.1)	25 (67.6)	4 (17.4)	102 (30.4)
*Smoking*				
Never	18 (34.0)	11 (29.7)	10 (43.5)	128 (38.0)
Ex-smoker	18 (34.0)	9 (24.3)	9 (39.1)	102 (30.4)
Current	16 (30.1)	15 (40.6)	4 (17.4)	102 (30.4)
Missing	1 (1.9)	2 (5.4)	0 (0.0)	4 (1.2)
*Total*	53 (100.0)	37 (100.0)	23 (100.0)	336 (100.0)

The occurrence of any SNEC was significantly related to ever-exposure to wood dust (OR = 11.4) in the age–sex-adjusted analyses (table 3). The risk for AD with wood dust exposure (OR = 58.6) was more than 10-fold that observed for OH, and these risk estimates were virtually unchanged after controlling for ever-exposure to other significant co-exposures (not shown). In table 4, ORs are presented for the final multivariable models, in which cumulative exposure was treated first as continuous and then as categorical variables (ordered exposure classes and ever exposed). The risk for AD more than doubled with every 5-year increase in exposure to wood dust (p<0.0001); for example, with 20 years of exposure the risk was 25 times higher than for those unexposed to wood dust after adjusting for co-exposures. A similar increasing effect was found on the risk of OH (p = 0.0001), but the dose–response relationship was less pronounced. A significant increase in the risk of AD (OR = 16.6) was also observed with ever-exposure at only low intensity (table 5).

**Table 3:** Odds ratios (ORs) for any sino-nasal epithelial cancer (SNEC) and three histological types with ever-exposure to occupational hazards, adjusted for age and sex

Hazard	All SNEC			Adenocarcinoma		Squamous cell carcinoma		Other histotypes	
	Cases (n = 113)	Controls (n = 336)	Adjusted odds ratios	Cases (n = 53)	Adjusted odds ratios	Cases (n = 37)	Adjusted odds ratios	Cases (n = 23)	Adjusted odds ratios
	No	No	OR (95% CI)	No	OR (95% CI)	No	OR (95% CI)	No	OR (95% CI)
Arsenic	6	7	2.2 (0.69 to 6.69)	1	0.64 (0.08 to 5.42)	3	5.2 (1.20 to 22.20)*	2	3.2 (0.59 to 17.26)
Wood dust	50	22	11.4 (6.29 to 20.74)***	41	58.6 (23.74 to 144.8)***	2	0.85 (0.19 to 3.83)	7	5.5 (1.99 to 15.24)***
Leather dust	9	2	14.4 (3.03 to 68.87)***	7	26.6 (5.09 to 139.0)***	1	5.0 (0.44 to 56.83)	1	6.3 (0.54 to 73.09)
Nickel	3	0		0		0		3	
Chromium	3	3	2.8 (0.55 to 14.06)	1	2.1 (0.22 to 21.1)	0		2	9.2 (1.40 to 60.13)*
PAHs	19	72	0.65 (0.37 to 1.15)	6	0.38 (0.15 to 0.94)*	9	1.2 (0.54 to 2.81)	4	0.66 (0.21 to 2.05)
Welding fumes	17	27	2.0 (1.00 to 3.82)*	6	1.3 (0.52 to 3.52)	9	4.1 (1.66 to 10.13)**	2	1.0 (0.22 to 4.66)
Oil mists	10	39	0.66 (0.32 to 1.40)	3	0.39 (0.11 to 1.33)	5	1.2 (0.43 to 3.39)	2	0.63 (0.14 to 2.84)
Formaldehyde	7	5	4.3 (1.32 to 14.10)*	6	9.5 (2.62 to 34.20)***	0		1	3.7 (0.38 to 36.53)
Flour dust	3	12	0.68 (0.19 to 2.48)	1	0.46 (0.06 to 3.68)	2	1.6 (0.34 to 7.43)	0	
Cocoa powder	1	1	4.2 (0.25 to 70.53)	0		1	9.6 (0.56 to 164.8)	0	
Silica	7	34	0.50 (0.21 to 1.19)	4	0.57 (0.19 to 1.70)	2	0.51 (0.12 to 2.28)	1	0.32 (0.04 to 2.52)
Coal dust	2	7	0.67 (0.14 to 3.32)	1	0.64 (0.08 to 5.42)	0		1	1.5 (0.17 to 13.0)
Textile dusts	12	31	1.4 (0.68 to 2.97)	6	1.9 (0.70 to 5.10)	2	0.52 (0.12 to 2.34)	4	2.8 (0.84 to 9.42)
Acid mists	6	10	1.7 (0.61 to 4.90)	2	1.3 (0.27 to 6.27)	0		4	6.3 (1.75 to 22.49)**
Paint mists	15	15	3.2 (1.47 to 6.76)**	11	5.3 (2.23 to 12.64)***	2	1.2 (0.26 to 5.46)	2	2.0 (0.43 to 9.75)
Organic solvents	44	42	4.3 (2.62 to 7.20)***	29	8.2 (4.32 to 15.72)***	5	1.1 (0.39 to 2.91)	10	5.7 (2.28 to 14.21)***

*p⩽0.05.

**p⩽0.01.

***p⩽0.001.

PAH, polycyclic aromatic hydrocarbon.

**Table 4:** Odds ratios (ORs) for any sino-nasal epithelial cancer (SNEC) and three histological types with cumulative exposure (CE) to occupational hazards, adjusted for co-exposures

All SNEC	CE treated as continuous variables							CE treated as categorical variables		
	OR (95% CI) at 5-year intervals over the range for which there were exposed cases and controls							Ordered exposure classes		Ever exposed
	5 years	10 years	15 years	20 years	25 years	30 years	35 years	OR* (95% CI)		OR* (95% CI)
Wood dust	1.77 (1.46 to 2.14)	3.1 (2.13 to 4.56)	5.5 (3.11 to 9.74)	9.7 (4.53 to 20.80)	17.1 (6.61 to 44.42)	30.2 (9.64 to 94.86)	53.4 (14.07 to 202.6)			
Leather dust	4.76 (1.21 to 18.73)†	22.7 (1.47 to 350.7)†						1–5 years	Over 5 years	14.3 (2.86 to 71.39)
								14.7 (1.37 to 156.7)	16.5 (1.75 to 154.7)	
Solvent vapours	1.27 (1.03 to 1.56)	1.6 (1.07 to 2.43)	2.0 (1.10 to 3.78)	2.6 (1.14 to 5.88)	3.3 (1.17 to 9.16)	4.2 (1.21 to 14.27)		1–10 years	Over 10 years	2.2 (1.17 to 3.97)
								1.4 (0.61 to 3.26)	3.5 (1.50 to 8.09)	
Welding fumes	1.34 (1.03 to 1.75)	1.8 (1.06 to 3.04)	2.4 (1.09 to 5.31)	3.2 (1.13 to 9.27)	4.3 (1.16 to 16.17)			1–10 years	Over 10 years	2.7 (1.31 to 5.45)
								2.4 (0.92 to 6.38)	3.0 (1.13 to 8.00)	
Arsenic			Not significant					1–10 years	Over 10 years	3.4 (1.03 to 11.43)
								1.6 (0.31 to 8.82)	9.3 (1.50 to 57.33)	
	**Adenocarcinoma**									
Wood dust	2.24 (1.76 to 2.85)	5.0 (3.09 to 8.10)	11.2 (5.42 to 23.04)	25.0 (9.53 to 65.55)	55.9 (16.74 to 186.5)	124.9 (29.4 to 530.7)	279.3 (51.67 to >999)			
Leather dust	6.94 (1.96 to 24.61)†	48.2 (3.84 to 605.4)†						1–5 years	Over 5 years	32.8 (5.96 to 181.1)
								11.3 (0.91 to 141.3)	58.1 (5.84 to 579.3)	
Solvent vapours	1.38 (1.07 to 1.78)	1.9 (1.15 to 3.16)	2.6 (1.24 to 5.61)	3.6 (1.33 to 9.97)	5.0 (1.42 to 17.72)	6.9 (1.53 to 31.48)		1–10 years	Over 10 years	4.3 (1.83 to 10.11)
								3.7 (1.28 to 10.89)	5.7 (1.82 to 18.00)	
Textiles	1.44 (1.08 to 1.94)	2.1 (1.16 to 3.77)	3.0 (1.24 to 7.31)	4.3 (1.33 to 14.18)	6.3 (1.43 to 27.53)	9.1 (1.54 to 53.42)		Not significant		
**Squamous cell carcinoma**										
Welding fumes	1.49 (1.12 to 1.98)	2.2 (1.25 to 3.94)	3.3 (1.40 to 7.81)	4.9 (1.56 to 15.50)	7.3 (1.75 to 30.76)			1–10 years	Over 10 years	3.7 (1.59 to 8.81)
								2.6 (0.69 to 9.46)	5.4 (1.87 to 15.33)	
Arsenic			Not significant					1–10 years	Over 10 years	4.4 (1.04 to 18.16)
								1.6 (0.16 to 15.55)	11.9 (1.61 to 88.07)	
**Other**	**histotypes**									
Wood dust‡	1.51 (1.22 to 1.87)	2.3 (1.49 to 3.48)	3.4 (1.82 to 6.48)	5.2 (2.22 to 12.09)	7.8 (2.70 to 22.54)	11.8 (3.30 to 42.03)	17.8 (4.02 to 78.37)			
Solvent vapours	1.36 (1.04 to 1.79)	1.9 (1.08 to 3.19)	2.5 (1.12 to 5.69)	3.4 (1.16 to 10.15)				1–10 years	Over 10 years	3.8 (1.45 to 10.09)
								2.6 (0.66 to 10.10)	6.2 (1.82 to 21.33)	
Acid mists	2.60 (1.35 to 5.01)	6.7 (1.81 to 25.11)	17.5 (2.44 to 125.8)					1–10 years	Over 10 years	7.5 (2.01 to 28.20)
								6.0 (1.32 to 26.98)	15.6 (1.30 to 186.8)	

*Adjusted for wood dust exposure on a continuous scale to reduce residual confounding (except for squamous cell).

†ORs are for 3.5 and 7 years, the latter being the maximum years of exposure among controls.

‡Exposure among cases ranged from 21 to 39 years.

**Table 5:** Odds ratios (ORs) for sino-nasal epithelial cancer (SNEC) by intensity of exposure to occupational hazards, adjusted for co-exposures

	Categorical intensity levels* (only low or ever medium–high)	
	Level	OR (95% CI)
**All SNEC**		
Wood dust	Low	3.2 (1.36 to 7.44)
	High	30.6 (11.93 to 78.30)
Leather dust	Low	17.6 (1.84 to 168.5)
	High	11.9 (1.12 to 127.1)
Solvent vapours	Low	2.3 (1.15 to 4.46)
	High	2.1 (0.65 to 6.67)
Welding fumes	Low	3.3 (1.47 to 7.26)
	High	1.6 (0.34 to 7.75)
Arsenic†	Ever exposed	4.1 (1.21 to 13.76)
**Adenocarcinoma**		
Wood dust	Low	16.6 (5.10 to 54.04)
	High	179.9 (55.37 to 584.4)
Leather dust	Low	52.4 (3.71 to 740.2)
	High	68.8 (5.60 to 844.7)
Solvent vapours	Low	4.5 (1.54 to 12.90)
	High	3.8 (0.67 to 21.58)
**Squamous cell carcinoma**		
Welding fumes	Low	3.5 (1.31 to 9.60)
	High	4.3 (1.01 to 18.10)
Arsenic†	Ever exposed	4.3 (1.01 to 18.10)
**Other histotypes**		
Wood dust	Low	2.1 (0.42 to 10.50)
	High	16.0 (3.93 to 65.24)
Solvent vapours	Low	4.8 (1.72 to 13.39)
	High	2.9 (0.33 to 24.73)
Acid mists	Low	5.6 (1.25 to 25.11)
	High	17.1 (1.42 to 206.7)

*Based on years of exposure weighted only by probability.

†Could not be categorised by intensity.

The age–sex-adjusted risk for any SNEC (OR = 14.4) with ever-exposure to leather dust was unchanged (OR = 14.3) after adjusting for wood dust and ever-exposure to other hazards (table 4), whereas the risk of AD (OR = 26.6) increased slightly (OR = 32.8) after controlling for wood dust and solvent vapours. A significant dose–response relationship was found between the risk of AD and years of leather dust exposure on a continuous scale (p = 0.003) and across ordered exposure classes (p<0.0002), with an almost 60-fold increase in risk with over 5 years’ exposure relative to unexposed. As with wood dust, low-intensity exposure significantly increased the risk for AD (OR = 52.4).

The significant twofold increase in the age–sex-adjusted risk for any SNEC with exposure to welding fumes was limited to SCC (OR = 4.1) and was essentially unchanged (OR = 3.7) after accounting for arsenic (OR = 4.4) (table 4). The risk of SCC increased by about 50% with every 5-year increase in exposure to welding fumes (p = 0.007). The trend in risk across ordered exposure classes was also significant (p = 0.001), as was the risk (OR = 3.5) with low-intensity exposure (table 5).

The age–sex-adjusted risk for any SNEC with ever-exposure to organic solvents (OR = 4.3) decreased but remained significant after controlling for exposure to wood dust and other hazards (OR = 2.2). A similar decrease was also observed in the age–sex-adjusted risks for AD and OH after controlling for co-exposures. The dose–response relationships found for AD and OH with increasing years of exposure to organic solvents on a continuous scale (p = 0.012 and p = 0.026) and across ordered exposure classes (p = 0.001 and p = 0.002) were also similar, as were the significantly increased risks with low-intensity exposure (OR = 4.5 and OR = 4.8, respectively). The slightly lower risk estimates at medium–high intensity were based on few exposed cases (table 5).

Ever-exposure to paint mists significantly increased the age–sex-adjusted risk for any SNEC (OR = 3.2) and, in particular, for AD (OR = 5.3), but its effect disappeared after accounting for exposure to organic solvents. Likewise, the relationship between any SNEC and formaldehyde exposure (OR = 4.3), also found for AD (OR = 9.5), lost significance after controlling for wood dust exposure.

The effect of chromium exposure on the risk of OH was significant in the age–sex-adjusted analysis (OR = 9.2), as was the association between OH and nickel exposure (Fisher’s exact test; p = 0.0002). A significant dose response with increasing years of exposure to strong acid mists was observed both on a continuous scale (p = 0.004) and across ordered exposure classes (p = 0.002) after controlling for co-exposures; however, three of the four cases exposed to acid mists had also been exposed to nickel or chromium, neither of which could be controlled for in the multivariable model (no exposed controls and insufficient number of exposed cases, respectively).

The relationship between AD and exposure to textile dusts was significant on a continuous scale (p = 0.015) with an increase in risk of over 40% with every 5-year increase in exposure. Aside from non-significant increased risks for any SNEC and SCC with exposure to cocoa powder, all other hazards produced non-significant risk estimates of less than 2 (table 3). Age, sex and smoking were not significant risk factors for SNEC or any histotype, after accounting for exposure to occupational hazards.

When the analyses were restricted to exposures that had occurred with at least 50% probability, the same final models were obtained for continuous exposure variables. Furthermore, the risk estimates for AD were very similar when those persons who had been exposed to leather dust were excluded (data not shown).

## DISCUSSION

The occurrence of any SNEC was significantly related to exposure to wood dust, leather dust, organic solvents, welding fumes and arsenic. The first three were significant predictors of AD, whereas exposure to welding fumes and arsenic were risk factors for SCC. Wood dust and organic solvents were also significantly related to OH, but the strength of the association with wood dust was one order of magnitude smaller than for AD. A significant effect of textile dust exposure on a continuous scale was also observed for AD.

Among the strengths of this study was the availability of accurate histological information, which meant that the risks for the two most common histological types, AD and SCC, could be investigated separately. Second, as all suspected occupational carcinogens were assessed with the exception of asbestos, the effects of co-exposures were considered. Exposure to asbestos, which has been associated with SNC^25^ and may be correlated with exposure to other hazards such as welding fumes, was not included because precision in reconstructing occupational exposure was considered unobtainable, especially for older working periods.

The association between SNEC and previous exposure to wood dust was confirmed and, specifically, with AD and OH for which dose–response effects were found. The increased risk for AD with low-intensity wood dust exposure appears to contradict the results from a pooled analysis of 12 case-control studies by Demers *et al*,^11^ but this discrepancy is likely explained by Demers’ reliance on a JEM to assign exposures, a method that usually results in substantial non-differential misclassification.^28^ In addition to the questionnaire responses, we referred to a JEM^25^ in assigning wood dust exposure, one in which intensity levels were defined by historical period, and low intensity corresponded to less than 1 mg/m^3^, medium intensity to 1–5 mg/m^3^ and high intensity above 5 mg/m^3^. It is unlikely that an exposure at or above 5 mg/m^3^, the occupational limit for wood dust in many countries including Italy, would have been rated as low intensity in the present study. Therefore, our finding suggests that the current exposure limit for wood dust should be considered for a substantial reduction, as proposed by the US National Institute of Occupational Safety and Health (NIOSH) as early as 1992.^29^ The absence of an association between SCC and wood dust exposure is consistent with the findings from other studies,^5^ ^19^ ^30^ although modestly increased risks with previous employment in woodworking have been reported.^22^ ^31^

The association between AD and leather dust exposure was also confirmed. The increasing risk of AD with years of cumulative exposure supports the existence of a dose–response relationship, also found in another case-control study conducted in Italy.^9^ Unlike wood dust, intensity levels for leather dust were not quantitative in the JEM^25^; nonetheless, the increased risk with low-intensity exposure suggests that workplace exposure to leather dust should also be minimised.

Several case-control studies in Nordic countries have reported an increased risk of SNC in occupations involving exposure to welding fumes,^20^ ^21^ ^32^ but none reported results stratified by histotype. In the present study, exposure to welding fumes increased the risk for SNEC and, in particular, for SCC with a dose–response relationship that, to our knowledge, is a new finding that supports a causal relationship and should be investigated further. Although this association can be confounded by exposure to nickel and chromium in stainless steel welding, none of the nine SCC cases exposed to welding fumes had ever been employed as stainless steel welders. In 1990, the IARC concluded that the evidence on welding fumes was limited and classified exposure to welding fumes as only possibly carcinogenic to humans (2B).^12^ Since then, exposure to welding fumes has also been linked to lung cancer in several epidemiological studies conducted in Europe and the USA, not only among stainless steel welders but also among mild steel welders and, in some instances, the risks increased with duration of exposure. A meta-analysis on the risk of lung cancer related to welding^33^ revealed significantly increased risks among both stainless and mild steel welders with similar relative risks, albeit the extent of confounding by asbestos exposure was not ascertained.

Among the other significant findings, the increased risks for AD and OH with exposure to organic solvents were similar in magnitude after controlling for other significant hazards, primarily wood dust. In the case of AD, the association may be attributable to residual confounding by wood dust owing to the strength of its effect, but very similar dose–response relationships were observed for both histogroups. This apparent association warrants further investigation, and as organic solvents include many chemicals, their individual effects should be examined. We are aware of only one report of an excess risk associated with organic solvents,^20^ and another of a significant association with paint, lacquer and glue, controlling for wood dust and formaldehyde.^34^

The significant dose response observed between the risk of AD and increasing textile dust exposure supports the existence of an association found in a pooled analysis of 12 case-control studies by Luce *et al*.^25^ This is also consistent with the few other studies that examined the risk separately by histotype,^4^ ^16^ although their findings were not significant.

Arsenic is another well-established carcinogen (group 1 IARC),^14^ but only a few case reports have been published on SNC and occupational exposure to arsenic.^35^ ^36^ To our knowledge, this is the first epidemiological study to report an association with arsenic, for which we observed a significant dose–response relationship. This finding requires verification, as risk estimates were based on few exposed cases.

The absence of an effect for exposure to formaldehyde contradicts the results of the pooled analysis by Luce *et al*,^25^ in which a significant association persisted after controlling for wood dust exposure. In a recent IARC monograph, formaldehyde was acknowledged as a definite human carcinogen, but evidence of an association with SNC was considered limited.^37^ No association was found between SNEC and smoking habits, in contrast with several studies that reported relative risks ranging from 1.2 to 3.0.^38^ In the present study, smoking habits could not be precisely defined as the information on intensity and duration was incomplete for many participants. Nevertheless, the smoking classification adopted (never, former, current smoker) has been shown to remove almost all confounding by smoking when assessing the effect of occupational exposures.^39^

Among the limitations of this study was the use of hospital controls, which may have distorted the risk estimates if exposure to occupational hazards among controls was not representative of the distribution in the general population. Another source of selection bias could have been the differential reporting of cases to the SNC Registry by hospitals more likely to notify the Registry of cases that had been exposed to the occupational hazards of interest, resulting in an overestimation of the risks. It is unlikely that this source of bias seriously affected our findings, as even among those cases reported to the Registry with probable or definite exposure to wood or leather dust, only 51% had been submitted for workers’ compensation (M Slaviero, personal communication, 2006). Moreover, incident cases were actively collected by the SNC Registry and the incidence rate in this study is comparable to that reported by the network of Italian Cancer Registries^40^ and higher than that in the Turin Registry (the largest in Piedmont), indicating good completeness in data collection. Another weakness of this study is the large number of cases excluded because an interview on occupational history was not available (15%), though there is no obvious reason why their exclusion would be related to prior exposure to occupational hazards. Furthermore, had all 20 excluded cases been unexposed to every hazard, this would have only slightly reduced the risk estimates for those exposures with low prevalence. Therefore, their exclusion is an unlikely explanation of our positive findings.

Recall bias may have been a source of differential misclassification of exposure, which would have produced an overestimation of the risks. The fact that interviewers were not blind to case-control status may also have distorted risk estimates if, for example, they had been more thorough in ascertaining exposure for cases than controls. Neither patients nor interviewers, however, had any prior knowledge of the suspected occupational risk factors, except perhaps for wood and leather dust. Moreover, the occurrence of SNEC was associated with only a few of the many hazards similarly characterised in the questionnaire by discernible exposure to fumes, dusts or vapours, which did not have any effect.

## CONCLUSIONS

Some occupational risk factors for SNEC (wood and leather dusts) were confirmed, and dose–response effects were observed for other hazards (welding fumes, organic solvents and textile dusts) that merit further investigation. The high risk for AD with low-intensity exposure to wood dust lends support for a reduction in the occupational threshold value. Many of the findings by histological type were based on few exposed cases and, therefore, the statistical adjustment for co-exposures may have been inadequate. The stability of the final models in the sensitivity analyses, however, strengthens our findings.
